# Health Tract, No. 200

**Published:** 1865

**Authors:** 


HEALTH TRACT, No. 200.

### WEATHER AND WEALTH.

"What has the weather to do with business?" was the reply of a cheery-faced and successful business man, to the inquiry: "Are you out such a day as this?" Such an hour of sleet and storm and angry howling winds is seldom seen in these latitudes. It was approaching three o'clock, and the bank account had to be made right, or financial ruin would have been the result. Suppose the storm had been ten times more tempestuous, the wind ten times more boisterous, the cold twenty degrees below zero, the City Hall clock would have struck three just as soon, and the bank notary would not have delayed one second later to have written the fatal word, "protested;" for business knows no law but that of promptitude; it knows no excuse; death even is no apology for the failure to meet a bank engagement. He who will succeed in making a fortune in a large city, must meet his engagements in all weathers.

It is precisely so in relation to health and disease. Moderate, daily exercise in the open air, with a cheerful spirit and an encouraging remuneration, is worth a thousand times more than all the remedies in the *materia medica* for the removal of ordinary ailments, when conjoined with temperance and cleanliness. But the same principle must be applied as in the successful prosecution of business. The exercise must be performed regardless of the weather. Not that exercise in bad weather is especially promotive to health; it is not as favorable to that end as good weather. But if exercise is needed at all, it is not the less necessary because it is raining, or very cold, or unendurably hot. If a man is hungry, he is not the less hungry because he can get nothing to eat. The necessity for exercise as a means of health is abiding; what makes the rule imperative, "Go out in all weathers" is, that we eat in all weathers; and if we exercise only when the weather is perfectly suitable, half the time would be lost in our changing climate. But the very energy and moral courage which enables a man to take out-door exercise, regardless of the weather, is of itself a potent means for the cure even of serious diseases.

The man who offers bad weather as an excuse for not going and paying a debt, will never succeed in business; nor will he get well, who, for that reason, fails to take his daily exercise, when it is an indispensable means of cure. It is precisely the same in religion; he who is swift to offer bad weather as an excuse for being absent from the worship of the great congregation on the Sabbath-day, or from other properly appointed "means of grace," never did make an efficient church member, will have nothing "added" in his napkin at the great accounting day! It is the man who is faithful to his duty, always, "regardless of the weather," or any thing else, who will hear the glad greeting from the Heavenly Judge, "Well done!"

